# *De novo *malignant solitary fibrous tumor of the kidney

**DOI:** 10.1186/1746-1596-6-96

**Published:** 2011-10-05

**Authors:** Tsan-Yu Hsieh, Yi-Che ChangChien, Wen-Hsiang Chen, Siu-Chung Chen, Liang-Che Chang, Cheng-Cheng Hwang, Hui-Ping Chein, Jim-Ray Chen

**Affiliations:** 1 Department of Pathology, Chang Gung Memorial Hospital, Keelung, Taiwan; 2 Department of Urology, Chang Gung Memorial Hospital, Keelung, Taiwan; 3 Department of Radiology, Chang Gung Memorial Hospital, Keelung, Taiwan; 4 College of Medicine, Chang Gung University, Tao-yuan, Taiwan

**Keywords:** solitary fibrous tumor, kidney, malignant, de novo, dedifferentiation, CD34

## Abstract

The kidney is a relatively infrequent site for solitary fibrous tumor (SFT). Among the previously reported cases, only two cases of malignant renal SFT developing via dedifferentiation from a pre-existing benign SFT have been reported. Here we reported a case of *de novo *malignant renal SFT clinically diagnosed as renal cell carcinoma in a 50-year-old woman. The tumor was circumscribed but unencapsulated and showed obvious hemorrhagic necrosis. Microscopically, the tumor was composed of patternless sheets of alternating hypercellular and hypocellular areas of spindle cells displaying mild to moderate nuclear atypia, frequent mitoses up to 8 per 10 high power fields, and a 20% Ki-67 proliferative index. Immunohistochemical studies revealed reactivity for CD34, CD99 and vimentin, with no staining for all other markers, confirming the diagnosis of SFT. No areas of dedifferentiation were seen after extensive sampling. Based on the pathologic and immunohistochemical features, a diagnosis of *de novo *malignant renal SFT was warranted. Our report expands the spectrum of malignant progression in renal SFTs. Even though this patient has been disease-free for 30 months, long-term follow-up is still mandatory.

## Backround

Solitary fibrous tumors (SFTs) are distinctive mesenchymal tumors most commonly described as pleural-based lesions; however they can develop at any extrapleural anatomic site [1]. Although the clinical course of SFTs is rather unpredictable, the prognosis of SFTs is generally favorable. It is estimated that 10% to 15% of intrathoracic SFTs and up to 10% of extrathoracic SFTs will recur and/or metastasize [2,3], therefore SFT is regarded as an "intermediate malignant, rarely metastasizing" neoplasm [4]. Microscopic features associated with malignancy in both intrathoracic and extrathoracic SFTs include nuclear atypia, increased cellularity and more than 4 mitoses per 10 high power fields [4,5]. An additional factor conferring a worse prognosis in SFTs is dedifferentiation or sarcomatous overgrowth, which represents an abrupt transition to a morphologically anaplastic component [6]. The kidney is a relatively infrequent site for SFT, with at least 36 cases reported in a review article [7]. The vast majority of renal SFTs are histologically benign and only two cases of malignant renal SFTs developing via dedifferentiation or sarcomatous overgrowth from a pre-existing benign SFT have been reported [7,8]. Here we report the first case of *de novo *malignant renal SFT without dedifferentiation and thus expand the spectrum of malignant progression in renal SFTs.

## Case presentation

### Clinical summary

**Figure 1:**
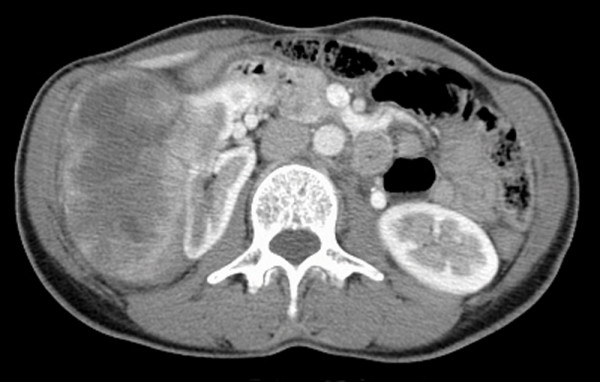
**CT of the abdomen**. Arterial phase images of dynamic computed topography scan showed a highly necrotic tumor compressing the renal parenchyma without either invasion to surrounding tissues or local lymphadenopathy.

A 50-year-old woman was admitted to our hospital with one-month history of soreness and pain in her right flank, without gross hematuria or other constitutional symptoms. Laboratory findings were unremarkable. Physical examination revealed a palpable right flank mass. A computed topography (CT) of the abdomen showed a huge necrotic tumor occupying the perirenal space of right kidney without evidence of either local invasion or lymphadenopathy (Figure 1). The patient underwent right radical nephrectomy under a pre-operative diagnosis of American Joint Committee on Cancer (AJCC) stage II (T2aN0) renal cell carcinoma. Post-operation course was smooth. Neither chemotherapy nor radiation therapy was given. She has been well without evidence of recurrence or metastasis for 30 months.

### Pathologic findings

**Figure 2:**
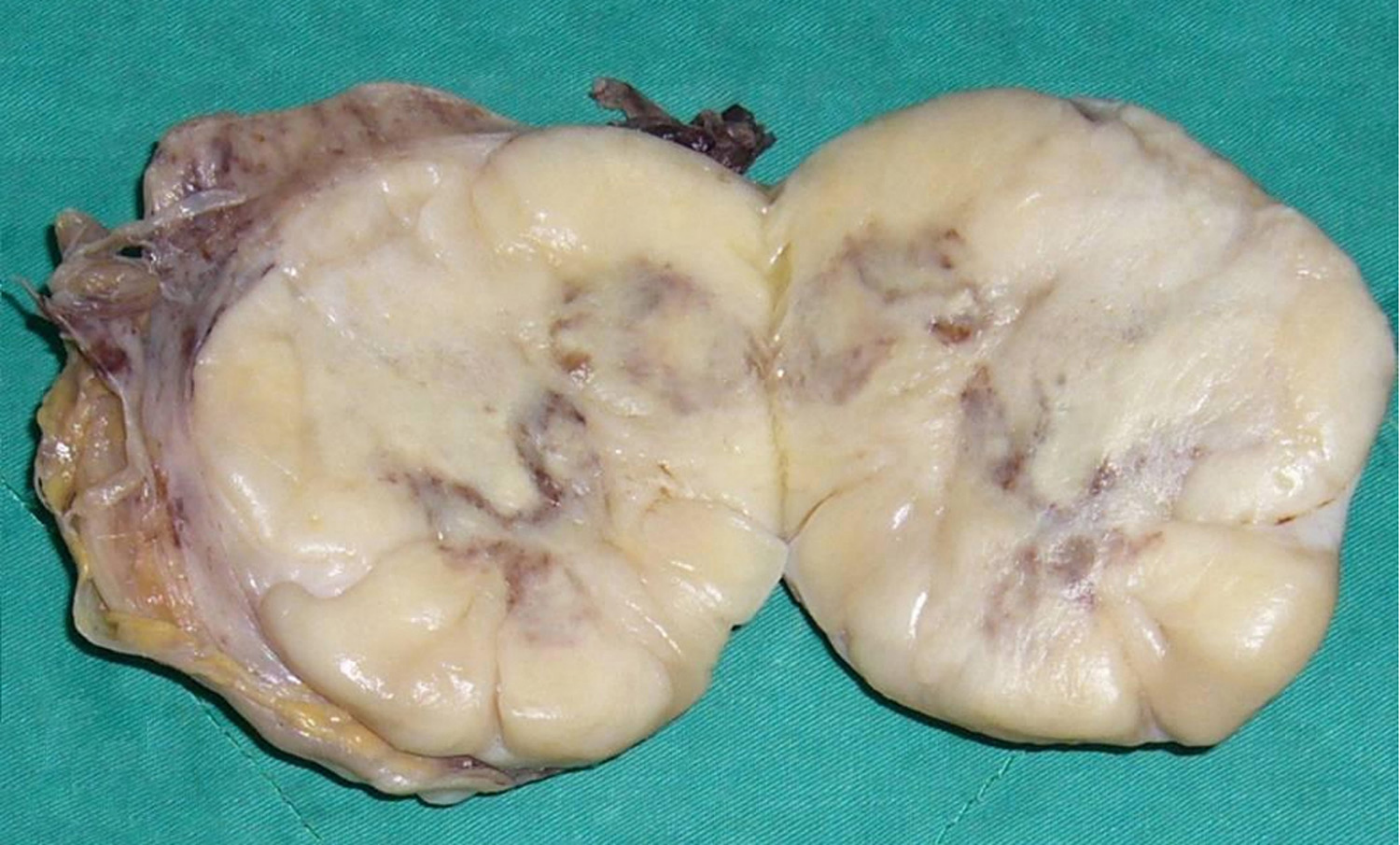
**Gross morphology**. The tumor was firm and showed a yellowish white to tan-gray, myxoid and lobulated cut surface with prominent hemorrhage and necrosis in the center.

**Figure 3:**
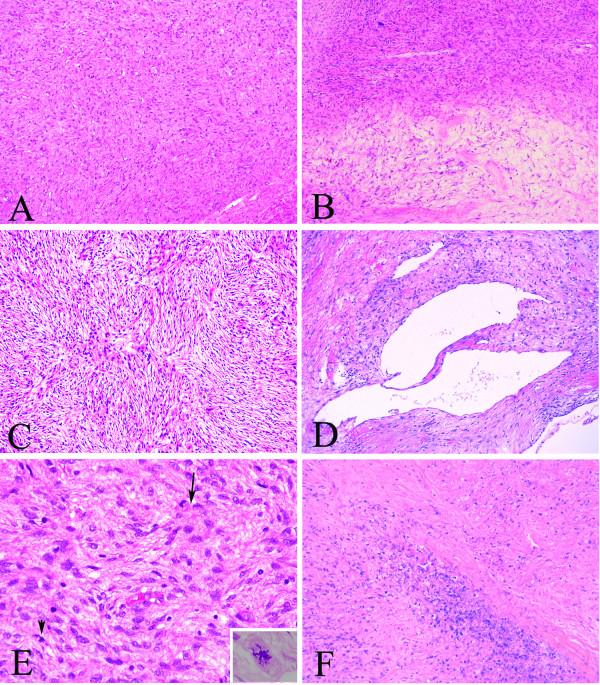
**Photomicrographs**. A, Proliferation of spindle cells arranging in a patternless architecture (×200 original magnification). B, Alternating hypercellular and hypocellular areas of spindle cells separated from each other by bands of collagen fiber (×200 original magnification). C, Spindle cells forming haphazard, storiform, or short fascicular arrangements in a loose myxoid to fibrous stroma containing dense collagen fibers (×200 original magnification). D, Hemangiopericytoma-like staghorn-like vessels (×200 original magnification). E, Tumor cells displaying mild to moderate atypia and 3 mitoses in this high power field (arrow and arrowhead) (×400 original magnification). Abnormal mitoses were occasionally seen (inset, ×400 original magnification). F, Prominent tumor necrosis (× 400 original magnification).

A nephrectomy specimen (15 × 9 × 7 cm, 670 g) with attached ureter and perirenal fibroadipose tissue was received. The specimen was bisected to reveal a 9 × 9 × 6 cm circumscribed but unencapsulated tumor occupying the perirenal space of the upper and middle poles of kidney. The tumor was firm and showed a yellowish white to tan-gray, myxoid and lobulated cut surface with prominent hemorrhage and necrosis (Figure 2). Microscopically, the tumor showed proliferation of spindle cells arranging in a patternless architecture (Figure 3A) with a combination of alternating hypercellular and hypocellular areas (Figure 3B). Haphazard, storiform, or short fascicular arrangements of spindle cells in a loose myxoid to fibrous stroma containing dense collagen fibers were also seen (Figure 3C). Dilated and branching hemangiopericytoma-like vessels were frequently observed (Figure 3D). Tumor cells had plump, fusiform, or elongated hyperchromatic nuclei with mild to moderate pleomorphism and indistinct cell borders and frequent mitoses up to 8 per 10 high power fields. Abnormal mitoses were occasionally seen (Figure 3E). Tumor necrosis was evidently present (Figure 3F). We did not find any areas of dedifferentiation after extensive tumor sampling.

**Figure 4:**
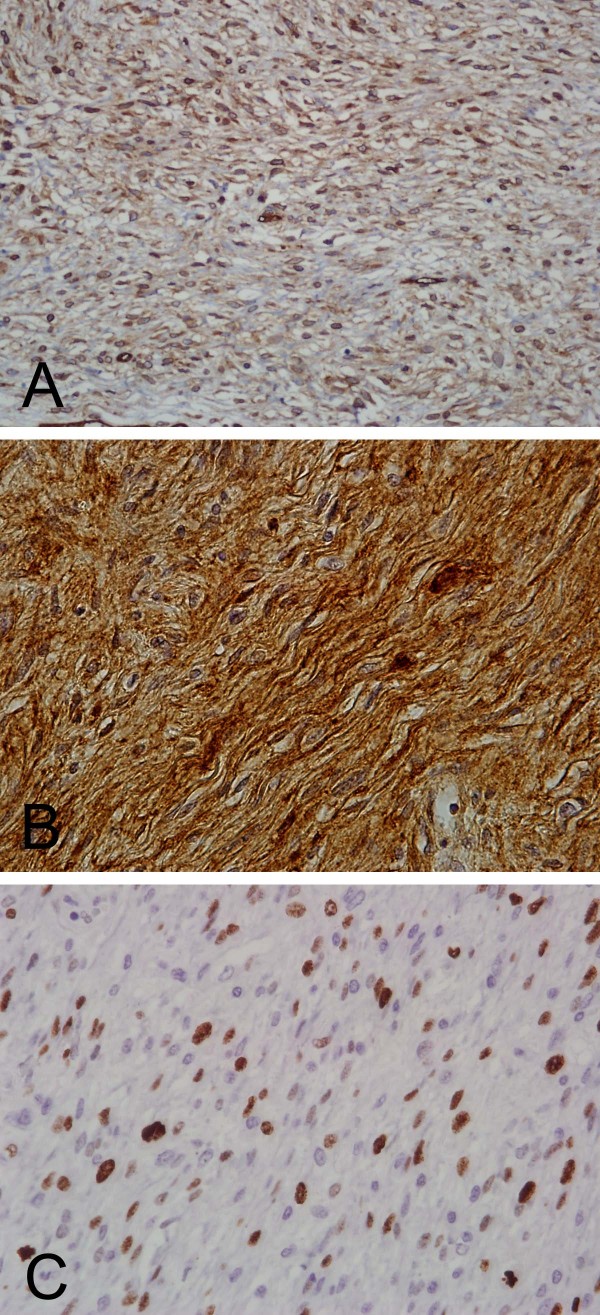
**Immunohistochemical photomicrographs**. Immunohistochemicaly, the tumor cells showing A, Weak to moderate CD34 immunostaining (×200 original magnification). B, Strong and diffuse CD99 immunostaining (×200 original magnification). C, Ki-67 immunostaining, analyzed by ImmuoRatio quantitative image software, showing a 20% proliferative index (×400 original magnification).

Immunohistochemically, the tumor showed weak CD34 positivity (Figure 4A) and diffusely strong CD99 (Figure 4B) and vimentin staining. They stained negatively for bcl-2 protein, S-100 protein, NSE, muscle markers, cytokeratin, CD117(C-kit), p53 and HMB-45. Ki-67 immunostaining, analyzed by ImmuoRatio quantitative image software [9], showed a 20% proliferative index. The immunohistochemical findings were highly consistent with SFT. Based on the microscopic features including increased cellularity, cellular atypia, frequent mitoses, a high proliferative index, necrosis and absence of dedifferentiation, a diagnosis of *de novo *malignant solitary fibrous tumor was established.

## Discussion

SFT is a relatively uncommon but distinctive mesenchymal neoplasm, originally described in the pleura cavity and later reported to occur ubiquitously [1]. Although the histogenesis of SFT remains undetermined, recent studies strongly favor a primitive mesenchymal or perivascular cell origin [10]. The kidney is a relatively infrequent site for SFT, with approximately at least 36 cases of renal SFT reported in a review article [7]. Clinically, these cases were frequently considered to be malignant due to their large tumor size by physical examinations and radiographic studies. Symptoms do not differ from those reported by patients with renal cell carcinoma. Hypoglycemia, which is a rare symptom in intrathoracic and extrathoracic SFTs, was not reported in any renal SFTs including our case [2].

Malignant behaviors in the form of recurrence and/or metastases can occur in 10% to 15% of intrathoracic SFTs and up to 10% of extrathoracic SFTs [2,3]. Malignant SFT is postulated to develop via two pathways: (1) *de novo *occurrence or (2) dedifferentiation or sarcomatous overgrowth from a pre-exsisting histologically benign SFT [1,4]. Most renal SFTs were classified as histologically benign and showed a favorable prognosis, with no evidence of recurrence during a follow-up period ranging from 2 to 89 months [7]. To our knowledge, two cases of malignant renal SFTs developing via dedifferentiation or sarcomatous overgrowth from a pre-existing benign SFT have been reported by Margo et al. and Fine et al., respecvievely [7,8]. A detailed comparison of the clinicopatholgoic features of these two cases and ours is summarized in Table 1. The tumor reported by Fine et al. had infiltrative borders and focal necrosis, and invaded beyond the renal capsule. The tumor described by Margo et al. was a 9-cm circumscribed mass devoid of either hemorrhage or necrosis. Notably, there was a 3-cm nodular area within the main tumor, microscopically responding to sarcomatous overgrowth. In contrast, our tumor showed a homogeneous cut surface with prominent necrosis and hemorrhage. Microscopically, the tumor reported by Fine et al. and Margo et al. showed typical features of benign SFT with 90% and 30% of dedifferentiation or sarcomatous overgrowth, respectively. In contrast, our tumor appeared to develop *de novo *since we did not find any areas of dedifferentiation after extensive tumor sampling.

**Table 1:** Comparsion of the clinicopathologic features of malignant renal solitary fibrous tumor

	**Fine et al**.	**Margo et al**.	Present case
Age (yr)	76	34	50

Gender	Female	Female	Female

Location	Left kidney	Left kidney	Right kidney

Size	12 × 10 × 7.5 cm	9 cm, with a distinct 3-cm nodule	9 × 9 × 6 cm

Tumor borders	Infiltrative with invasion beyond the renal capsule	Circumscribed	Circumscribed, unencapsulated

Necrosis	Focal	Absent	Marked

Hemorrhage	NA	Absent	Present

Histology	10% benign SFT 90% dedifferentiation	70% benign SFT 30% dedifferentiation	*De novo *malignant SFT

Mitoses	Frequent	2-6/10 HPF	8/10 HPF

Atypia	Marked in areas of dedifferentiation	Marked in areas of dedifferentiation	Mild to moderate

Ki-67 proliferative index	NA	1-4%	20%

CD34	Loss of expression in areas of dedifferentiation	Positive	Positive

Follow-up	Multiple lung nodules 4 month after nephrectomy	15 months, NED	30 months, NED

NA: not available, NED: no evidence of disease

The criteria for clinical malignancy in intrathoracic SFT, first proposed by England et al. in 1989, include increased cellularity, pleomorphism, mitotic count more than 4 per 10 high power fields, necrosis, hemorrhage, size more than 10 cm, non-pedunculated and atypical locations (parietal pleura, pulmonary parenchyma) [5]. The diagnostic criteria for malignant extrathoracic SFTs are purely microscopic and include increased cellularity, pleomorphism and mitotic count more than 4 per 10 high power fields [4,11]. Currently the impact of tumor characteristics (size, hemorrhage, necrosis and location) in predicting clinical malignancy in extrathoracic SFTs remains to be investigated. Our case fulfilled the diagnostic criteria for malignant extrathoracic SFTs. Additionally, the presence of hemorrhage and necrosis and a 20% Ki-67 proliferative index further supports a diagnosis of malignant renal SFT.

SFTs show a wide variety of microscopic growth patterns and should be distinguished from benign and malignant spindle cell tumors. Positive immunoreactivity for CD34 and CD99 is characteristic of SFT, and highly valuable in differentiating from other mesenchymal tumors [2,4]. However the expression of CD34 and CD99 may be decreased or absent in areas with marked atypia or dedifferentiation [8,12]. Genetic analyses of SFT to date have not found consistent and characteristic cytogenetic abnormalities that can be used an ancillary diagnostic marker. Missense mutation of platelet-derived growth factor receptor-β (PDGFR-β) has been reported in 2 of 88 pleuro-pulmonary SFTs [13], but we did not detect PDGFR-β mutation in our case (data not shown).

The prognosis of extrapleural SFTs is more unpredictable than that of pleural tumors due to lack of large-scale studies. The clinical outcomes were rather strikingly different in the two malignant renal SFTs with dedifferentiation. While a patient developed multiple small lung nodules 4 months post-operatively [8], the other patient was disease-free after 15 months of follow-up [7]. Our patient has been well without evidence of recurrence or metastasis for 30 months. Some studies indicated that extrapleural SFTs had similar prognosis as the pleural counterpart, and a tumor with malignant histological feature was associated with recurrence and metastasis [3,14], but other studies showed that extrapleural SFTs tended to have a favorable outcome than pleural ones, even those with malignant histological features [11,15]. Studies also showed that deep-seated locations, over-expression of p53 and p16, loss of CD34 immunostaining and the presence of dedifferentiated areas may indicate more aggressive behaviors [6]. Complete surgical excision and long-term follow-up are generally recommended for patients with extrapleural SFTs [15]. Besides, due to the rich vascularity and possible origin from pericytes, antiangiogenic therapy, especially for the advanced cases is also under clinical investigation [16].

## Consent

Written informed consent was obtained from the patient for publication of this case report and accompanying images. Submission of this case report was approved by Institutional Review Board (IRB) of Chang Gung Memorial Hospital, Tao-Yuan, Taiwan. A copy of the written consent is available for review by the Editor-in-Chief of this journal.

## List of abbreviations

SFT: solitary fibrous tumor

## Competing interests

The authors declare that they have no competing interests.

## Authors' contributions

TYH was responsible for data collection, literature search and manuscript preparation. YCCC carried out gross and microscopic examinations. WHC performed the surgery and clinical follow-up of the patient. CSC interpreted pre-operative and follow-up imaging studies. CLC, CCH and HPC participated in the microscopic analyses and helped making a final diagnosis. JRC participated in manuscript preparation and approved the final manuscript. All authors read and approved the final manuscript.
